# The control of magma crystallinity on the fluctuations in gas composition at open vent basaltic volcanoes

**DOI:** 10.1038/s41598-020-71667-7

**Published:** 2020-09-10

**Authors:** Julia Woitischek, Marie Edmonds, Andrew W. Woods

**Affiliations:** 1 Department of Earth Sciences, University of Cambridge, Downing St, Cambridge, CB2 3EQ UK; 2 BP Institute, University of Cambridge, Madingley Rd, Cambridge, UK

**Keywords:** Solid Earth sciences, Geochemistry, Geophysics, Petrology, Volcanology

## Abstract

Basaltic open vent volcanoes are major global sources of volcanic gases. Many of these volcanoes outgas via intermittent Strombolian-type explosions separated by periods of passive degassing. The gas emitted during the explosions has high molar CO_2_/SO_2_ and SO_2_/HCl ratios, while during the passive degassing these ratios are lower. We present new laboratory experiments in a model volcanic conduit, which suggest that these differences in gas geochemistry are a consequence of gas migration through crystal-rich magma. We show that gas may flow along channels through the particle-laden liquid and, at a critical depth, the gas may displace an overlying crystal-rich plug *en masse*, producing a growing slug of gas. Owing to the friction on the walls of the conduit, this plug becomes progressively sheared and weakened until gas enriched in the least soluble volatiles breaks through, causing an explosion at the surface. When the gas slug bursts, liquid is drawn up in its wake, which exsolves the more soluble volatile components, which then vent passively at the surface until the next explosive slug-bursting event.

## Introduction

**Figure 1:**
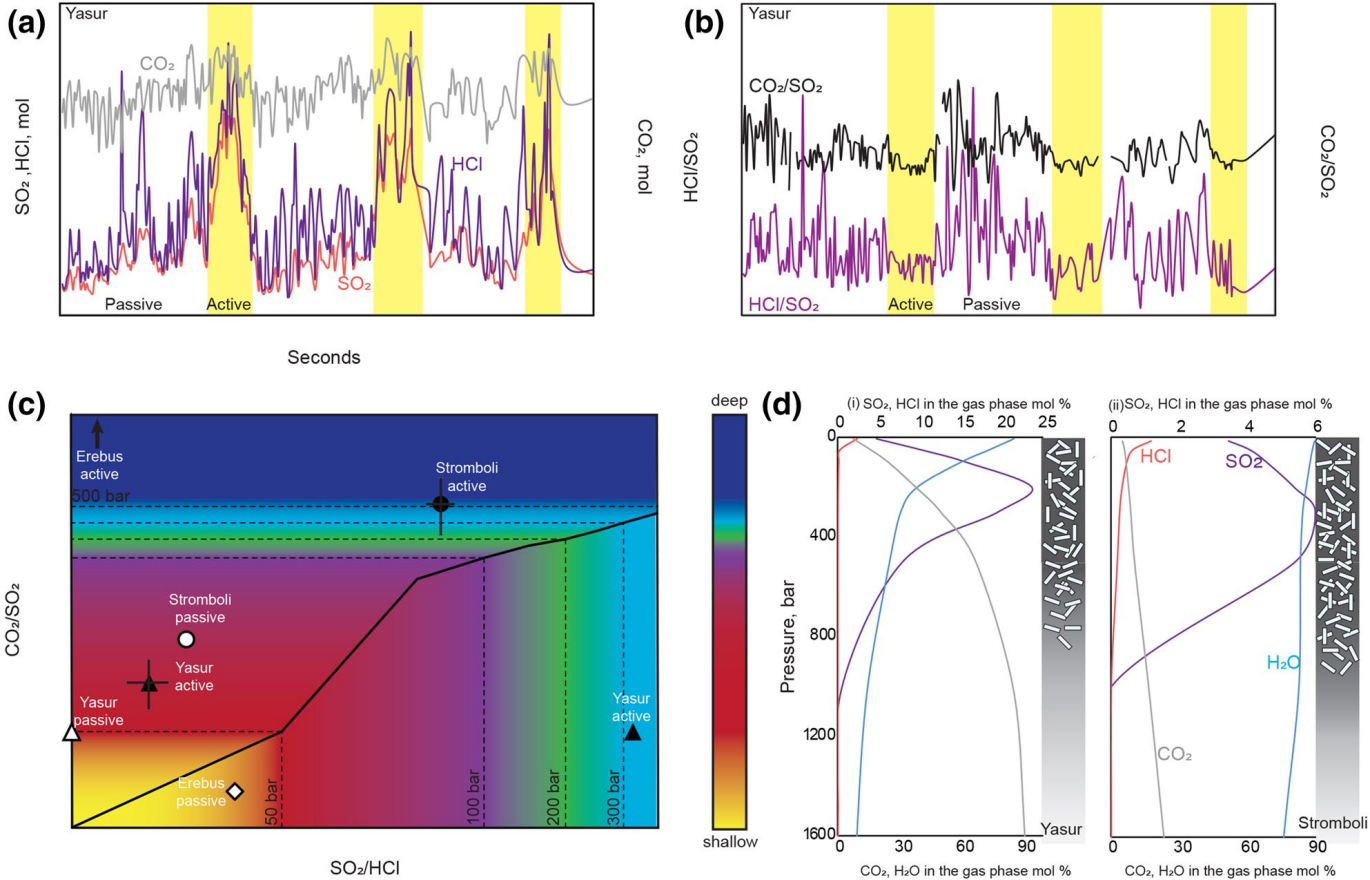
Bimodal volcanic gas signatures associated with active and passive degassing regimes at basaltic open vent volcanoes reveal both shallow and deeper conduit sources. (**a**) Variation in the surface measurements of CO_2_, SO_2_ and HCl over 300 s at Yasur Volcano are caused by Strombolian-type explosions (yellow rectangle)^5^. (**b**) At Yasur Volcano, the molar HCl/SO_2_ ratio during active degassing periods is low whereas during passive periods it is higher^5^. (**c**) Molar CO_2_/SO_2_ plotted against molar SO_2_/HCl for volcanic gases emitted during active periods (black symbols) compared to passive periods (white symbols) for a Stromboli^2^ (circles), Erebus^4, 18^ (diamonds) and Yasur^1, 5^ (triangles) volcanoes. The standard deviation of the data is shown by vertical and horizontal bars. The evolution of gas composition with pressure is shown for Stromboli (black solid line) for different pressures (dashed lines)^21^. (**d**) The variation of the gas composition (in mol %) in terms of CO_2_ (grey line), SO_2_ (violet line), H_2_O (blue line) and HCl (orange line) in the magma during closed degassing as the pressure varies from 1,600 bar to the surface at Yasur and Stromboli volcanoes^21^. An interpretive illustration of the shallow conduit is drawn next to each gas exsolution evolution diagram. Stromboli’s crystal-rich zone may extend deeper in the conduit than Yasur’s crystal-rich plug, which would be consistent with the higher molar CO_2_/SO_2_ ratio during active degassing at Stromboli (see text for discussion and supporting arguments).

It is well known that volcanic gases emitted from volcanoes that exhibit Strombolian-type eruptions (e.g. Yasur, Stromboli, Villarrica, Erebus) are characterised by cyclic variations in volcanic gas flux and composition^1–5^, with the bursting of large bubbles called slugs (‘active’ degassing) generating gases with relatively high molar CO_2_/SO_2_ and SO_2_/HCl ratios, and degassing between explosions (‘passive’ degassing) generating gases with lower ratios (Fig. 1a–c). It has been proposed that these cycles in observed volcanic gas composition are associated with separated bubble-magma flow coupled with either magma degassing at different depths in the conduit^6^ or with the intermittent release of gas slugs from a foam layer deep in the system if gas accumulates at the top of a sill^7, 8^. However, petrological studies have shown that the magma erupted from these volcanoes is highly crystalline, with > 30 vol.% crystals in the upper few hundred metres below the surface, with the crystallisation largely driven by water exsolution^9–12^. The presence of crystals may be a key control on the migration of gas bubbles through the magma. Previous studies show that bubble growth and coalescence processes are directly affected by the amount and size of particles (crystals) in a liquid: at low particle contents, the bubbles are small and round^13^ whereas at higher particle fractions, bubbles may become trapped and change their shape. At a high magma crystallinity, the gas may migrate through the magma along series of fractures or channels^13–17^.

## Chemical variations in gas emission at volcanoes exhibiting Strombolian activity

In complementary studies, it has been shown that gas emitted from Stromboli^2^, Yasur^1, 5^ and Erebus^4, 18^ volcanoes are characterised by bimodal compositions as quantified by the ratios of CO_2_/SO_2_ and SO_2_/HCl (Fig. 1a–c). Figure 1a,b show that during Strombolian-type eruptions (active degassing) at Yasur, a series of intermittent explosions emit gases with an increased molar ratio of SO_2_/HCl. These explosions are separated by periods of passive degassing during which the gases released at the surface have lower molar SO_2_/HCl ratios (Fig. 1a,b). A similar behaviour can be observed at Stromboli and Erebus volcanoes, which shows an even stronger variation between the molar CO_2_/SO_2_ ratio during active and passive degassing (caused principally by the higher initial CO_2_ content in their magma, Fig. 1c)^2, 4, 18^. At Stromboli, passive degassing is more enriched in HCl than active degassing^2^ whereas during both phases of activity at Erebus the molar SO_2_/HCl ratios are similar ^4, 18^.

The ratios of emitted volcanic gases can be used to estimate gas equilibration depths with some knowledge of their solubility behaviour. The solubilities of volatiles in the magma depends on temperature, pressure and magma composition^19–21^ and the widely accepted sequence of solubility in basaltic melts is CO_2_ < S < Cl^22^. Since CO_2_ exsolves at higher pressures than S and Cl, it has been concluded by many authors that large bubbles causing Strombolian eruptions have a deeper origin compared to bubbles causing passive degassing, which are more enriched in HCl^8^ (Fig. 1b). Figure 1d shows a representative equilibrium gas exsolution model^21^ for magma with a composition similar to that of Yasur and Stromboli volcanoes to show the geochemical evolution of the gas phase exsolving from the melt during decompression from 1,600 bar to the surface at a constant temperature of 1,150 °C in a closed and equilibrium degassing mode. In Fig. 1c, the black solid line represents the CO_2_/SO_2_ and SO_2_/HCl gas exsolution line for Stromboli and the curves in Fig. 1d, panel ii are given for CO_2_, SO_2,_ H_2_O and HCl in the gas phase for Stromboli. The combination of the measured changes in CO_2_/SO_2_ and SO_2_/HCl ratios at the surface (Fig. 1a–c) and the gas exsolution models (Fig. 1d) suggest that the bimodal gas compositions during ‘typical’ degassing activity originate from two depths, with a deep bubble origin (Stromboli^2^: 600–150 bar; Yasur^5^: 180–360 bar, Erebus^23^: 420–2,100 bar; Fig. 1d) during active Strombolian periods and a shallow bubble source (Stromboli^2^: 75–0 bar; Yasur^5^: 120–0 bar; Erebus^23^: 420–0 bar) for passive periods. We propose that this type of bimodal gas geochemistry is typical of open vent basaltic volcanoes that exhibit Strombolian behaviour and below, we link these trends to interactions between gases and crystal-rich magmas in the shallow plumbing system.

## The effect of high magmatic crystallinity on degassing behaviour

**Figure 2:**
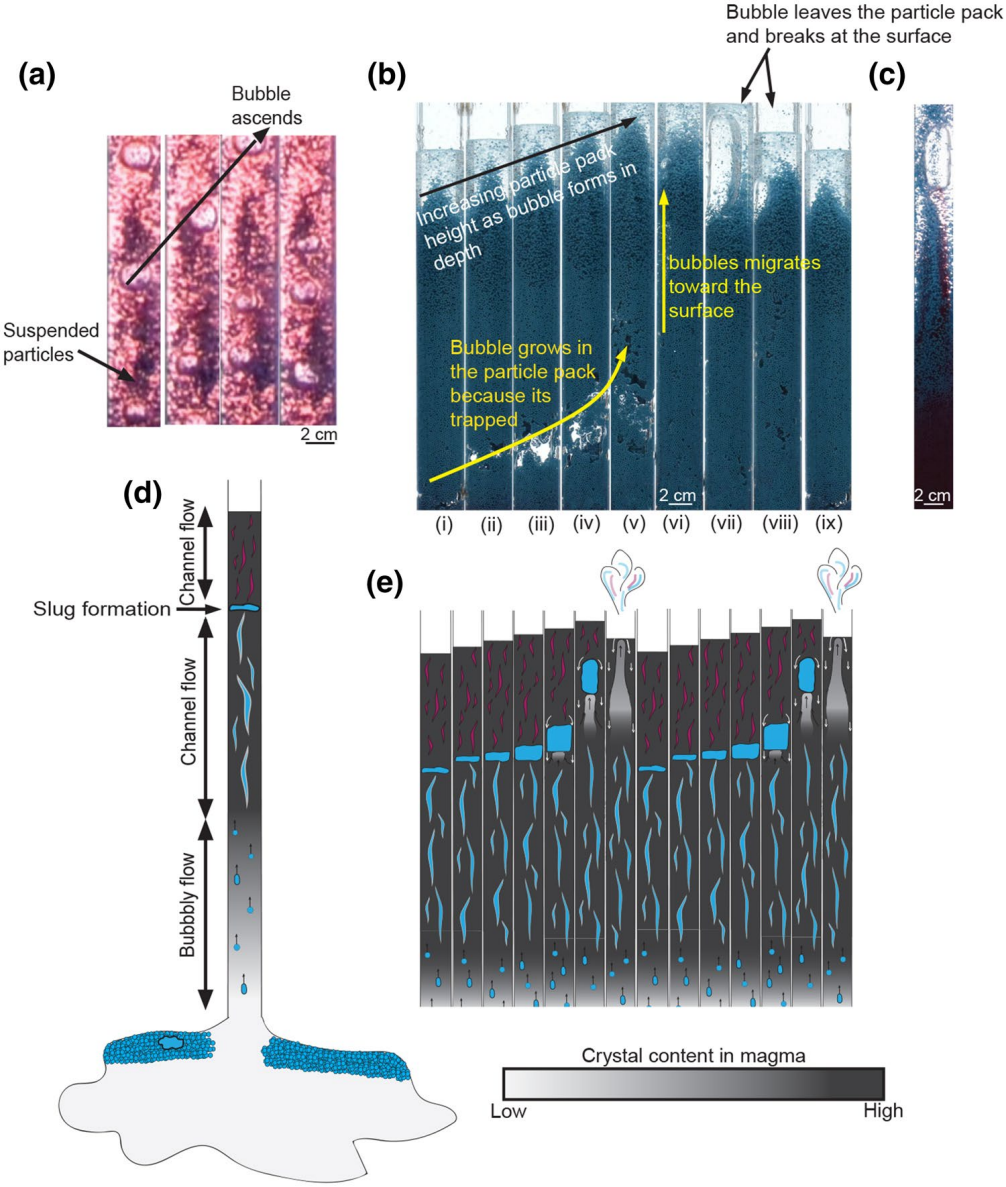
Analogue experiments reveal the presence of particles may cause intermittent bursting of slugs interspersed by passive degassing regimes. (**a**) Photos of a low particle content experiment (particle fraction ≤ 0.1) in a vertical tube illustrating the frequent ascent of smaller bubbles in a liquid with suspended particles. (**b**) Time series of images from a high particle load experiment showing the formation, migration and bursting of a gas slug. Panel (i) to (vii): Image taken every second. (**c**) Image showing the transport of coloured liquid in the wake of a slug as it rises through the particle-rich mixture*.* Slug moves on a timescale of 10 s whereas liquid moves on a timescale of 1,000 s, which might lead to the shallow exsolution of dissolved gas from the magma. (**d**,**e**) Illustration showing channel flow in the deeper conduit and intermittent growth, ascent and explosions of the gas slugs, shaded blue, giving rise to the active degassing periods and channel flow near the surface giving rise to passive degassing, shaded purple . The crystal content in the magma is illustrated by the grey colour scale. All panels are described in the text.

To study the influence of a high crystal concentration on magma degassing, we develop an analogue experimental model in which we use a mixture of water, glycerol and particles (with a diameter of 0.002 m) in a 2 m long vertical tube, with a radius of 0.02 m (Table 1). We compare experiments in which the particles occupy 10% to more than 40% of the volume of the liquid-particle mixture. We supply a steady flux of gas in the range 10^–3 ^–7 × 10^–3^ l s^−1^ and observe the migration of the gas through the particle-laden liquid (Fig. 2). In the experiments, for convenience, we allowed the particles to settle to the base of the tube prior to adding the gas flux. As we report, with a thin particle zone, the particles mixed throughout this liquid layer to form a low-particle content liquid; with the higher gas fluxes, the particles did not mix to the top of the liquid layer, but remained as a high particle-content zone with a pure liquid layer above. This liquid layer did not appear to influence the processes in the underlying particle layer, but allowed for easy observation of the gas leaving the top of the particle-laden layer.

**Table 1:** Experimental parameters. A systematic increase of particle load allowed for between 24 and 27 experiments for each experimental set-up (A–D).

Experimental set-up	A	B	C	D
Viscosity (Pa s)	1.0	0.8	0.4	0.2
Gas flux 1 (l s^−1^)	10^–3^	10^–3^	10^–3^	10^–3^
Gas flux 2 (l s^−1^)	4 × 10^–3^	4 × 10^–3^	4 × 10^–3^	4 × 10^–3^
Gas flux 3 (l s^−1^)	7 × 10^–3^	7 × 10^–3^	7 × 10^–3^	7 × 10^–3^
Particles	The mass of particles was systematically increased from 73 to 1818 g in increments of 73 g of particles; the viscosity and gas flux remained constant in each case.

In the low particle load experiment, we find that small bubbles rise continuously and form a dilute particle suspension, as seen in Fig. 2a^6–8, 24^. In contrast, in the experiments in which there is a large particle load in the liquid, we find that a particle suspension does not develop and instead, the gas migrates upwards through small channels in the packed layer of  particles, as has been reported by^15–17^. However, in the present experiments, we also find that on reaching a depth of about 0.4 to 0.5 m below the top of particle layer (corresponding to particle volume fractions of 40 to 60%), a gas slug steadily grows below the overlying particle plug, which as a result, is displaced upwards. This growing slug causes the overall level of the liquid-particle mixture to increase (Fig. 2b, panels i-iv). Eventually, the plug above the growing slug is weakened by the internal deformation associated with the frictional stress from the walls of the conduit, and the slug of gas breaks through to the surface (Fig. 2b, panels vi–viii) where the gas is emitted and the process resumes (Fig. 2b, panel ix). During gas ascent, some of the deeper liquid is drawn up in the wake of the gas, exchanging with the liquid originally above the slug (Fig. 2b, panel vii, 2c).

## Transition from channel flow to slug formation

**Figure 3:**
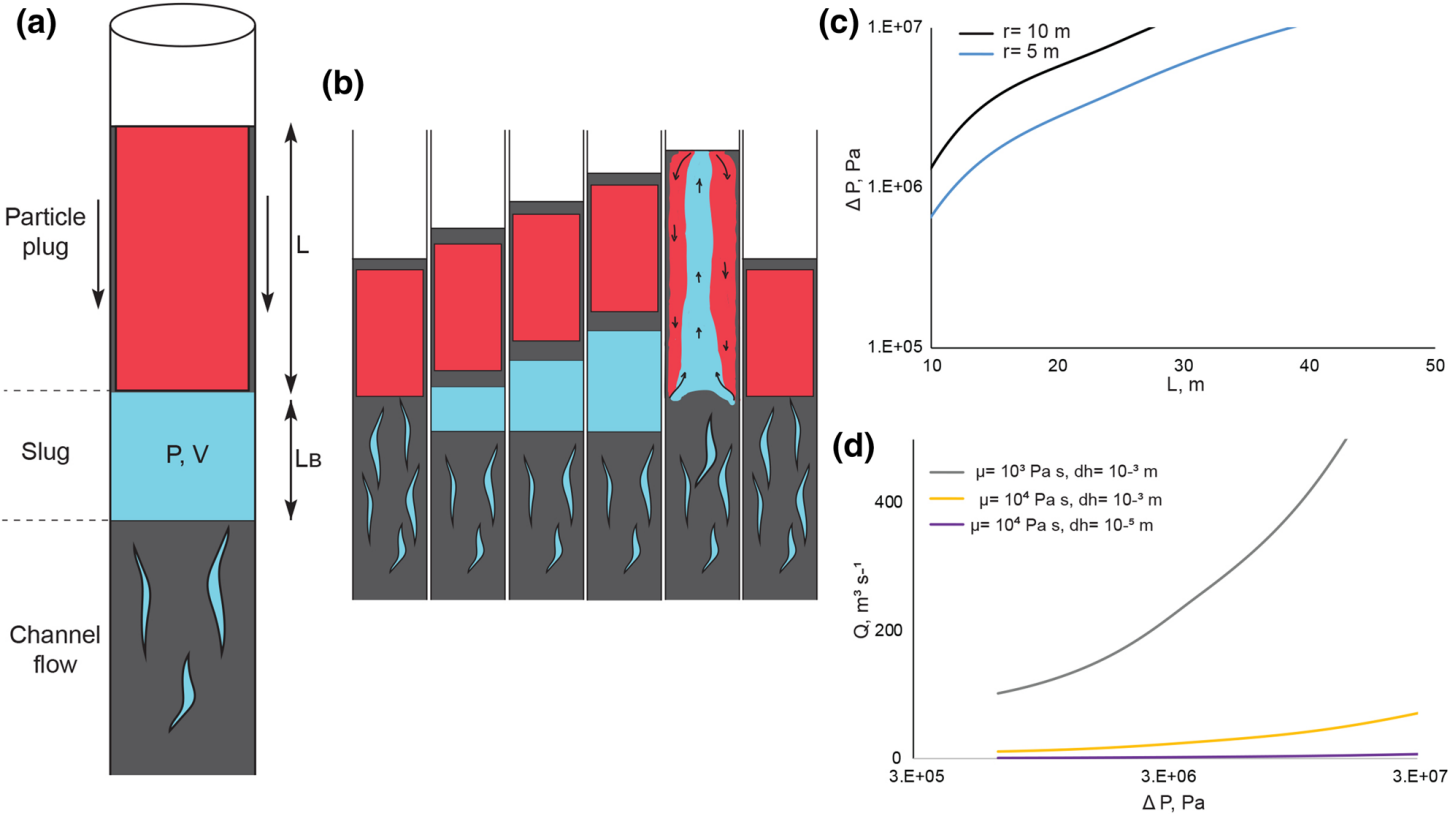
Simplified shallow volcanic conduit showing our model for Strombolian eruptions. (**a**,**b**) Simplified illustration of the volcanic conduit showing channel flow, a slug and a particle plug as well as the cycle of slug growth, plug weakening, slug bursting and plug reformation, as discussed in text. (**c**) The length of the particle plug, L, is plotted as a function of the gas overpressure, ΔP for a vent radius, r, equal to 10 and 5 m. (**d**) The relation between the gas flux, Q, and ΔP. For a given gas flux, the dynamic overpressure is smaller for a magma with larger value of the lubrication layer d_h_ or lower viscosity (grey line).

We may rationalise the behaviour described above for the high particle load experiments, in that the particles become close packed and develop an effective frature strength^25–29^. The supply pressure of the gas that is supplied at the base of the system, is able to overcome this  strength and form a local channel in the pack along which the gas can flow. In the lower part of the particle pack, the supply pressure is smaller than the sum of the weight of the overlying column of liquid and particles (i.e. the column-static pressure) plus the static friction resisting the bulk displacement of the overlying particle pack along the walls. However, as the gas migrates upwards, the depth of the overlying particle pack becomes progressively smaller, and so both (i) the column-static pressure and (ii) the static friction suppressing the motion of the overlying particle pack decrease. Eventually at a critical depth, the gas is able to displace the overlying particle pack and there is a transition from channel flow to bulk displacement of the overlying particle pack. This leads to formation of a gas slug as further gas continues to be supplied from the deeper channel flow and accumulates in the slug (Fig. 3).

Once the particle plug starts moving, the overpressure determines the frictional resistance of the particle plug on the walls of the conduit. This wall stress also leads to gradual deformation of the particle plug as it moves upwards. Eventually the plug loses its strength, enabling the gas to break through to the surface. The process then repeats.

The depth at which the gas can form a slug is given by the balance between the gas overpressure ΔP, which is given by the fracture strength of the particle pack, the coefficient of static friction on the walls, λ, and the normal stress on the conduit walls, integrated from the slug to the surface of the crystal-magma plug, leading to the balance in Eq. (1). We have integrated the product of the normal stress on the wall of the conduit over the area of the crystal plug. In doing this we require a model of how the overpressure of the bubble is dissipated across the plug, since on the upper surface of the plug the pressure is atmospheric. For simplicity, we assume the overpressure ΔP leads to a linear pressure gradient through the plug which produces a part of the normal stress on the wall of the conduit, the remainder being associated with the magmastatic pressure; the static frictional stress is equal to the fraction λ of this normal stress. Just before the plug begins to move, the vertical force associated with the overpressure on the base of the crystal plug matches this frictional stress:

1 $$\Delta\ Pr ^{2} \pi = 2\pi r\lambda \left( {\frac{{\Delta {\text{PL}}}}{2} + \frac{{\rho {\text{gL}}^{2} }}{2}} \right)$$

where r is the radius of the particle plug, L is the height of the particle plug, ρ is the density of the liquid-particle mix and g is the gravitational acceleration. This balance determines particle plug size, L, for a given overpressure. Using the parameters for our experiments, and the observation that L = 0.5 m, Eq. (1) suggests that at the onset of motion, ΔP has a value of order of 10 Pa, which is consistent with earlier laboratory measurements^26, 27^ .

Subsequently, the plug begins to move upwards and the system adjusts so that the overpressure matches the stress on the walls. The speed of the particle pack is constrained by the gas flux, Q, from depth. If the motion of the plug is lubricated by liquid at the walls of the conduit, the vertical growth of the gas slug in the particle pack grows upwards with the speed, u, and has overpressure, ΔP, given by:

2 $${\text{u}} = {Q \mathord{\left/ {\vphantom {Q {\left( {r^{2} \pi } \right) = \frac{{d_{h} }}{2\pi rL\mu }\pi r^{2} \Delta P}}} \right. \kern-\nulldelimiterspace} {\left( {r^{2} \pi } \right) }}$$

3 $$\frac{2\pi rL\mu u}{{d_{h} }} = \Delta \ Pr^{2} \pi$$

where d_h_ represents the thickness of the lubricating fluid layer at the edge of the conduit, which we expect to scale with the diameter of the particles, and µ is the viscosity of the melt.

In our experiments, we find that u = 0.01 m s^−1^ and so we estimate that ΔP = 10 Pa, using the value µ = 0.23–1.0 Pa s, which is appropriate for the glycerol-water mixture at room temperature and assuming that the boundary layer thickness d_h_ is of order 0.001 m comparable to the size of the particles.

As the slug rises and breaks through to the surface, we observe that some liquid is drawn up in the wake of the slug, thereby exchanging fluid between the upper particle plug and fluid deeper in the conduit (Fig. 2c). After the release of the gas, a particle plug reforms at the top of the conduit, and the process repeats (Fig. 3b).

Although the model is highly simplified, and does not simulate all the complexities of the observations of Strombolian eruptions^30–33^, it does illustrate some of the episodicity, which may arise when gas migrates through a crystal-rich liquid. It is nonetheless of interest to explore the magnitude of the model predictions using parameters for a volcanic system. In Fig. 3c, we show the relation between ΔP and the length of the plug, L, (Eq. (1),(2)) and find that for a magma fracture strength of order 10^5^–10^6^ Pa, the model predicts a plug length L in the range 10–30 m if the conduit has a radius of order 5–10 m. In Fig. 3d, we show the overpressure as a function of the rate of growth of the slug as given by Eq. (3). We find that for gas fluxes of 1–500 m^3^ s^−1^, the overpressure required to drive the plug to the surface is of order ~ 10^5^–10^6^ Pa if the lubricating layer has thickness d_h_ of order 0.03–0.001 m, consistent with the size of the crystals in the melt, assuming the magma has a viscosity in the range of 10^3^–10^4^ Pa s. We note that in a volcanic conduit, the frictional resistance may also be influenced by the roughness of the conduit walls, which may span a comparable range of length scales.

At Stromboli Volcano, we expect the magma to have a viscosity of 4,400 Pa s^34^, a conduit radius of about 3 m^35^ and a crystal size of 0.001 m^10^. With these parameters, our model suggests that the length of the plug is of order 10–50 m and with a gas flux in the range 0.4–1.9 m^3^ s^-1^ the eruption period is about 13 min. Moreover, the plug which is filled with crystal-rich magma with a crystal content of about 50 vol. % ^10^ (composition in appendix Table 3) could store as much as 8–38 m^3^ of gas. Depending on the rate of gas-flow through the plug, some of this could then issue from the vent during the passive degassing phase. For Yasur Volcano, if we use a viscosity of 6,722 Pa s, a conduit radius of 4 m and a crystal size of 0.005 m ^9^, the model suggests that the plug length is in the range 10–25 m and that with a gas flux in the range 4–10 m^3^ s^-1^ the dynamic overpressure is similar to the overpressure at the onset of the motion of the plug. This leads to an eruption period of about 2 min. These simple estimates of eruption frequency at Stromboli and Yasur are comparable to those reported in previous studies^2, 5, 30–33^. Yasur’s plug is filled with a crystal-rich^9^ magma containing 32 vol. % of crystals (composition in appendix Table 3). This could be host to as much as 6–16 m^3^ of gas, some of which may issue during the passive degassing phase. This total mass is an upper bound on the mass of gas which may be present in the plug owing to the separation of the gas from the melt deeper in the conduit. However, these values illustrate that there is a significant volume of gas available for exsolution from the melt, and some of this may be associated with the passive degassing phase of the eruption cycles.

## Conclusion

The range of phenomena in our experiments has some analogy with that observed at volcanoes that exhibit Strombolian activity. When the crystal content is high, between 30 and 60 vol.% we observe intermittent explosions interspersed with passive degassing behaviour. In contrast, with smaller particle concentrations, the flow more closely resembles a steadily bubbling liquid column leading to much smaller temporal fluctuations in flow and hence in mean gas composition (Fig. 2a).

Turning back to the field data on gas composition, we suggest that bubbles accumulating in the slug reflect the CO_2_ and SO_2_ signature of gas ascending from greater depths so that typical Strombolian explosions have gas with a relatively high molar CO_2_/SO_2_ and SO_2_/HCl ratio. In contrast, the magma transported to shallow depths in the wake of the slug degasses at low near-surface pressures, consistent with depths of 10–50 m, leading to passive venting of gas with a low molar CO_2_/SO_2_ and SO_2_/HCl ratio, as seen in Fig. 1c.

This new picture of Strombolian activity in a high crystal content magma is consistent with observations at other volcanoes and it would be interesting to collect more high frequency data on the gas geochemistry to provide further insights into the processes that control Strombolian eruptions.

## Supplementary information

Supplementary file1

Supplementary file2

Supplementary file3
