# Supplementary material for: The control of magma crystallinity on the fluctuations in gas composition at open vent basaltic volcanoes
Source: Sci Rep. 2020 Sep 10;10:14862. doi: 10.1038/s41598-020-71667-7 (PMC7483529; doi:10.1038/s41598-020-71667-7)
Supplement: Supplementary file 2 — Supplementary file2 [file 41598_2020_71667_MOESM2_ESM.pdf]

Supplementary Material
The control of magma crystallinity on the fluctuations in gas composition at open vent basaltic volcanoes

J Woitischek, M Edmonds, A.W. Woods

Table 1 Molar fraction of volcanic gases (excluding H_2O) emitted from Yasur Volcano in 350 seconds shown in 236 measurements, Vanuatu during active and passive phases in July 2018. The sample abbreviation can be understood as sampling date and time as follows: year_month_day_hour and minute_seconds[5]

sample	*SO_2*	*HCl*	*CO_2*	***sample***	*SO_2*	*HCl*	*CO_2*
18_07_16_0641_49	0.10	0.11	0.79	18_07_16_0645_38	0.10	0.22	0.68
18_07_16_0641_51	0.06	0.07	0.87	18_07_16_0645_40	0.07	0.11	0.82
18_07_16_0641_53	0.19	0.21	0.61	18_07_16_0645_41	0.24	0.19	0.57
18_07_16_0641_54	0.07	0.10	0.83	18_07_16_0645_43	0.10	0.09	0.81
18_07_16_0641_56	0.07	0.08	0.85	18_07_16_0645_46	0.11	0.43	0.45
18_07_16_0641_57	0.07	0.14	0.79	18_07_16_0645_48	0.10	0.18	0.72
18_07_16_0641_59	0.09	0.17	0.74	18_07_16_0645_49	0.11	0.30	0.59
18_07_16_0642_00	0.15	0.30	0.55	18_07_16_0645_52	0.12	0.15	0.73
18_07_16_0642_02	0.08	0.12	0.81	18_07_16_0645_54	0.09	0.16	0.75
18_07_16_0642_04	0.09	0.15	0.76	18_07_16_0645_56	0.12	0.20	0.68
18_07_16_0642_05	0.09	0.11	0.79	18_07_16_0645_57	0.09	0.15	0.76
18_07_16_0642_07	0.07	0.14	0.79	18_07_16_0645_59	0.09	0.18	0.73
18_07_16_0642_08	0.08	0.10	0.82	18_07_16_0646_02	0.30	0.31	0.39
18_07_16_0642_10	0.10	0.14	0.77	18_07_16_0646_05	0.09	0.23	0.68
18_07_16_0642_11	0.08	0.10	0.82	18_07_16_0646_08	0.10	0.27	0.64
18_07_16_0642_13	0.06	0.08	0.85	18_07_16_0646_10	0.06	0.08	0.86
18_07_16_0642_15	0.15	0.25	0.61	18_07_16_0646_11	0.05	0.14	0.81
18_07_16_0642_16	0.06	0.08	0.87	18_07_16_0646_14	0.10	0.18	0.72
18_07_16_0642_18	0.06	0.13	0.81	18_07_16_0646_16	0.11	0.22	0.67
18_07_16_0642_21	0.23	0.46	0.31	18_07_16_0646_17	0.09	0.14	0.77

18_07_16_0642_22	0.07	0.14	0.79	18_07_16_0646_22	0.08	0.12	0.80
18_07_16_0642_25	0.09	0.12	0.79	18_07_16_0646_24	0.08	0.13	0.80
18_07_16_0642_29	0.15	0.19	0.66	18_07_16_0646_25	0.09	0.09	0.82
18_07_16_0642_30	0.10	0.13	0.77	18_07_16_0646_27	0.08	0.13	0.78
18_07_16_0642_32	0.08	0.09	0.82	18_07_16_0646_28	0.13	0.15	0.72
18_07_16_0642_33	0.15	0.18	0.66	18_07_16_0646_30	0.08	0.15	0.77
18_07_16_0642_36	0.43	0.49	0.07	18_07_16_0646_33	0.10	0.17	0.73
18_07_16_0642_38	0.08	0.15	0.76	18_07_16_0646_36	0.09	0.14	0.77
18_07_16_0642_44	0.12	0.13	0.75	18_07_16_0646_39	0.08	0.12	0.80
18_07_16_0642_46	0.18	0.19	0.64	18_07_16_0646_41	0.07	0.14	0.79
18_07_16_0642_47	0.14	0.21	0.65	18_07_16_0646_43	0.09	0.15	0.76
18_07_16_0642_49	0.14	0.17	0.69	18_07_16_0646_44	0.08	0.14	0.79
18_07_16_0642_51	0.21	0.79	0.00	18_07_16_0646_47	0.11	0.11	0.78
18_07_16_0642_52	0.12	0.18	0.69	18_07_16_0646_49	0.12	0.13	0.76
18_07_16_0642_54	0.13	0.22	0.65	18_07_16_0646_50	0.08	0.13	0.79
18_07_16_0642_57	0.15	0.24	0.61	18_07_16_0646_52	0.13	0.24	0.63
18_07_16_0642_58	0.11	0.15	0.74	18_07_16_0646_53	0.08	0.13	0.78
18_07_16_0643_00	0.17	0.27	0.56	18_07_16_0646_55	0.10	0.15	0.75
18_07_16_0643_02	0.11	0.21	0.68	18_07_16_0646_57	0.09	0.18	0.73
18_07_16_0643_03	0.12	0.12	0.76	18_07_16_0646_58	0.18	0.32	0.50
18_07_16_0643_05	0.15	0.16	0.69	18_07_16_0647_00	0.10	0.17	0.73
18_07_16_0643_06	0.09	0.20	0.71	18_07_16_0647_01	0.13	0.13	0.75
18_07_16_0643_08	0.12	0.20	0.68	18_07_16_0647_05	0.10	0.15	0.75
18_07_16_0643_09	0.11	0.22	0.67	18_07_16_0647_06	0.12	0.10	0.78
18_07_16_0643_12	0.15	0.39	0.46	18_07_16_0647_08	0.12	0.14	0.74
18_07_16_0643_16	0.16	0.17	0.67	18_07_16_0647_09	0.14	0.11	0.75
18_07_16_0643_19	0.11	0.18	0.71	18_07_16_0647_11	0.17	0.12	0.70
18_07_16_0643_20	0.19	0.17	0.63	18_07_16_0647_12	0.16	0.15	0.69
18_07_16_0643_22	0.11	0.11	0.78	18_07_16_0647_14	0.17	0.19	0.64
18_07_16_0643_24	0.08	0.15	0.77	18_07_16_0647_15	0.15	0.21	0.64

18_07_16_0643_25	0.10	0.11	0.79	18_07_16_0647_17	0.20	0.21	0.59
18_07_16_0643_28	0.08	0.09	0.83	18_07_16_0647_20	0.15	0.16	0.69
18_07_16_0643_30	0.14	0.18	0.68	18_07_16_0647_23	0.14	0.17	0.69
18_07_16_0643_33	0.08	0.09	0.83	18_07_16_0647_26	0.14	0.18	0.68
18_07_16_0643_36	0.09	0.12	0.79	18_07_16_0647_31	0.17	0.21	0.62
18_07_16_0643_38	0.10	0.09	0.81	18_07_16_0647_33	0.18	0.19	0.63
18_07_16_0643_39	0.09	0.16	0.75	18_07_16_0647_34	0.15	0.13	0.72
18_07_16_0643_41	0.14	0.14	0.72	18_07_16_0647_36	0.20	0.18	0.63
18_07_16_0643_42	0.13	0.22	0.65	18_07_16_0647_37	0.23	0.20	0.57
18_07_16_0643_44	0.10	0.12	0.78	18_07_16_0647_39	0.16	0.16	0.68
18_07_16_0643_47	0.10	0.10	0.80	18_07_16_0647_42	0.13	0.17	0.71
18_07_16_0643_49	0.10	0.17	0.74	18_07_16_0647_45	0.14	0.22	0.63
18_07_16_0643_50	0.15	0.22	0.62	18_07_16_0647_47	0.15	0.20	0.64
18_07_16_0643_52	0.09	0.10	0.81	18_07_16_0647_48	0.15	0.22	0.63
18_07_16_0643_53	0.14	0.17	0.69	18_07_16_0647_50	0.16	0.16	0.67
18_07_16_0643_55	0.10	0.19	0.71	18_07_16_0647_55	0.13	0.14	0.73
18_07_16_0643_56	0.18	0.17	0.65	18_07_16_0648_07	0.35	0.65	0.00
18_07_16_0643_58	0.12	0.12	0.77	18_07_16_0648_09	0.13	0.17	0.70
18_07_16_0644_00	0.10	0.16	0.73	18_07_16_0648_20	0.12	0.31	0.57
18_07_16_0644_03	0.11	0.17	0.73	18_07_16_0648_23	0.17	0.23	0.60
18_07_16_0644_06	0.11	0.19	0.70	18_07_16_0648_26	0.17	0.31	0.53
18_07_16_0644_07	0.16	0.25	0.59	18_07_16_0648_28	0.10	0.18	0.72
18_07_16_0644_09	0.14	0.22	0.64	18_07_16_0648_29	0.10	0.22	0.68
18_07_16_0644_11	0.18	0.18	0.63	18_07_16_0648_31	0.09	0.18	0.73
18_07_16_0644_12	0.13	0.14	0.72	18_07_16_0648_32	0.10	0.10	0.80
18_07_16_0644_14	0.10	0.18	0.72	18_07_16_0648_34	0.14	0.14	0.71
18_07_16_0644_15	0.10	0.18	0.72	18_07_16_0648_35	0.11	0.12	0.77
18_07_16_0644_18	0.09	0.10	0.81	18_07_16_0648_39	0.14	0.09	0.77
18_07_16_0644_20	0.14	0.20	0.66	18_07_16_0648_40	0.16	0.16	0.68
18_07_16_0644_21	0.12	0.17	0.72	18_07_16_0648_42	0.23	0.22	0.55

18_07_16_0644_23	0.18	0.25	0.57	18_07_16_0648_45	0.12	0.17	0.71
18_07_16_0644_26	0.12	0.16	0.72	18_07_16_0648_48	0.09	0.15	0.76
18_07_16_0644_29	0.12	0.15	0.72	18_07_16_0648_51	0.15	0.16	0.69
18_07_16_0644_31	0.16	0.18	0.66	18_07_16_0648_54	0.17	0.17	0.67
18_07_16_0644_32	0.18	0.20	0.63	18_07_16_0648_57	0.11	0.15	0.74
18_07_16_0644_34	0.17	0.22	0.62	18_07_16_0648_59	0.10	0.18	0.72
18_07_16_0644_36	0.18	0.18	0.64	18_07_16_0649_00	0.09	0.13	0.78
18_07_16_0644_37	0.17	0.19	0.64	18_07_16_0649_04	0.12	0.13	0.74
18_07_16_0644_39	0.16	0.18	0.66	18_07_16_0649_05	0.15	0.12	0.73
18_07_16_0644_40	0.16	0.21	0.63	18_07_16_0649_08	0.15	0.28	0.57
18_07_16_0644_42	0.22	0.23	0.55	18_07_16_0649_11	0.33	0.26	0.41
18_07_16_0644_43	0.17	0.17	0.66	18_07_16_0649_15	0.15	0.21	0.63
18_07_16_0644_45	0.17	0.20	0.63	18_07_16_0649_18	0.13	0.27	0.60
18_07_16_0644_47	0.16	0.19	0.65	18_07_16_0649_21	0.09	0.08	0.83
18_07_16_0644_48	0.20	0.27	0.53	18_07_16_0649_22	0.09	0.08	0.83
18_07_16_0644_50	0.16	0.19	0.65	18_07_16_0649_26	0.09	0.18	0.73
18_07_16_0644_51	0.20	0.27	0.53	18_07_16_0649_35	0.14	0.19	0.67
18_07_16_0644_53	0.15	0.20	0.64	18_07_16_0649_36	0.10	0.11	0.79
18_07_16_0644_54	0.19	0.18	0.64	18_07_16_0649_40	0.07	0.15	0.78
18_07_16_0644_56	0.18	0.25	0.57	18_07_16_0649_43	0.08	0.17	0.75
18_07_16_0644_58	0.13	0.17	0.69	18_07_16_0649_44	0.08	0.20	0.71
18_07_16_0644_59	0.14	0.18	0.68	18_07_16_0649_48	0.08	0.12	0.80
18_07_16_0645_01	0.12	0.16	0.73	18_07_16_0649_5	0.11	0.13	0.76
18_07_16_0645_02	0.11	0.14	0.75	18_07_16_0649_52	0.11	0.12	0.77
18_07_16_0645_04	0.12	0.11	0.77	18_07_16_0649_54	0.13	0.20	0.67
18_07_16_0645_05	0.11	0.15	0.74	18_07_16_0649_55	0.12	0.16	0.72
18_07_16_0645_07	0.26	0.32	0.42	18_07_16_0649_57	0.12	0.13	0.75
18_07_16_0645_08	0.09	0.15	0.76	18_07_16_0650_00	0.15	0.14	0.71
18_07_16_0645_12	0.10	0.15	0.75	18_07_16_0650_02	0.14	0.21	0.65
18_07_16_0645_15	0.06	0.07	0.87	18_07_16_0650_05	0.15	0.14	0.71

18_07_16_0645_16	0.06	0.06	0.88	18_07_16_0650_06	0.17	0.22	0.61
18_07_16_0645_18	0.07	0.08	0.86	18_07_16_0650_08	0.16	0.13	0.70
18_07_16_0645_19	0.06	0.17	0.78	18_07_16_0650_09	0.15	0.21	0.64
18_07_16_0645_23	0.05	0.06	0.88	18_07_16_0650_11	0.14	0.23	0.63
18_07_16_0645_26	0.37	0.52	0.11	18_07_16_0650_13	0.19	0.15	0.66
18_07_16_0645_29	0.10	0.20	0.70	18_07_16_0650_14	0.15	0.13	0.72
18_07_16_0645_30	0.05	0.09	0.86	18_07_16_0656_03	0.14	0.12	0.74
18_07_16_0645_32	0.05	0.09	0.86	18_07_16_0656_47	0.07	0.10	0.83
18_07_16_0645_35	0.07	0.21	0.72				

data for figure 1a and b in main paper

Table 2 Mean molar ratios of active and passive phases measured at Erebus[4,17], Stromboli[2] and Yasur[1,5]. Some mean ratios are given with the standard deviation. n represents the approximate number of all measurements including active and passive degassing phases

**Volcano**	*n*	*Active*		*Passive*	
		CO_2/SO_2	SO_2/HCl	CO_2/SO_2	SO_2/HCl
Erebus	546	169 ± 83	1.18 ± 0.24	1.30	2.68
Stromboli	28880	10.4 ± 1.04	9.40 ± 1.6	3.90	2.00
*Yasur**	477	2.85 ± 0.25	1.5 ± 0.20	1.96 ± 0.20	0.5 ± 0.30
Yasur	2800	2	30	2	1

Data for figure 1c in the main text

Erebus: Passive gas data from [4] were measured by OP-FTIR during 4200 seconds in December 2004 and a spectra was collected every 8 seconds. Active data were measured by [17] and we used the average gas composition of 21 explosive events of type bubble I and II in 2005, 2006 and 2013.
Stromboli: Active and passive data measured by [2] with OP-FTIR during 11,520 seconds in 2002 with a time temporal resolution of 4 seconds.
Yasur:* S/Cl ratios from table 1 measured during 241 seconds in 2018; CO_2/S ratios for active and passive were measured during 222 seconds and 477 seconds, respectively in 2018 (Woitischek et al., in review).
Yasur: Passive and active phases was measured in 2006 by [1] with OP-FTIR over 2480 seconds.

Table 3 Melt inclusion composition of Stromboli and Yasur in wt. %.

**Volcano**	*SiO_2*	*Al_2O_3*	*CaO*	*K_2O*	*Na_2O*	*MgO*	*FeO*	*H_2O*	*CO_2*	*S*	*Cl*
Stromboli	*48.24*	*15.43*	*15.19*	*1.71*	*2.46*	*4.28*	*6.97*	*3.0*	*2.0*	*0.25*	*0.22*
Yasur	*49.53*	*16.94*	*10.04*	*1.09*	*3.22*	*4.85*	*9.03*	*1.0*	*0.30*	*0.09*	*0.05*

Data used in [20] for figure 1d in the main text

Stromboli was measured by [9], the CO_2 composition is from [36]
Stromboli was measured by [8], the CO_2 composition is from [5]

Additional Reference

36 Aiuppa, S. et al. A model of degassing for Stromboli volcano. *Earth Planet. Sci. Lett*. **295**, 195-204 (2010).
