# Supplementary material for: The control of magma crystallinity on the fluctuations in gas composition at open vent basaltic volcanoes
Source: Sci Rep. 2020 Sep 10;10:14862. doi: 10.1038/s41598-020-71667-7 (PMC7483529; doi:10.1038/s41598-020-71667-7)
Supplement: Supplementary file 3 — Supplementary file3 [file 41598_2020_71667_MOESM3_ESM.docx]

Video

Video 1 **Typical evolution of an crystal-rich experiment**
